# Digital Device Usage and Childhood Cognitive Development: Exploring Effects on Cognitive Abilities

**DOI:** 10.3390/children11111299

**Published:** 2024-10-27

**Authors:** Vicente Javier Clemente-Suárez, Ana Isabel Beltrán-Velasco, Silvia Herrero-Roldán, Stephanie Rodriguez-Besteiro, Ismael Martínez-Guardado, Alexandra Martín-Rodríguez, Jose Francisco Tornero-Aguilera

**Affiliations:** 1 Faculty of Sport Sciences, European University of Madrid, 28670 Villaviciosa de Odón, Spain; vctxente@yahoo.es (V.J.C.-S.); stephanie.rodriguez@universidadeuropea.es (S.R.-B.); josefrancisco.tornero@universidadeuropea.es (J.F.T.-A.); 2 Grupo de Investigación en Cultura, Educación y Sociedad, Universidad de la Costa, Barranquilla 080002, Colombia; 3 Psychology Department, Faculty of Life and Natural Sciences, Nebrija University, 28240 Madrid, Spain; abeltranv@nebrija.es; 4 Faculty of Applied Social Sciences and Communications, International Business University, UNIE, 28015 Madrid, Spain; silvia.herrero@universidadunie.com; 5 LFE Research Group, Department of Health and Human Performance, Faculty of Physical Activity and SportScience (INEF), Universidad Politécnica de Madrid, Calle de Martín Fierro, 7, 28040 Madrid, Spain; imartinezgu91@gmail.com

**Keywords:** digital device usage, childhood cognitive development, attention, memory, executive functions, psychological resilience, educational technology

## Abstract

The increasing ubiquity of digital devices in childhood had outpaced the understanding of their effects on cognitive development, creating a significant research gap regarding their long-term impact. Objective: The present narrative overview explored the complex relationship between digital device usage and cognitive development in childhood. Methods: We conducted a comprehensive literature search across multiple databases, including PubMed, Embase, Scopus, and Web of Science, to critically assess cognitive domains such as attention, memory, executive functions, problem-solving skills, and social cognition. Incorporating over 157 peer-reviewed studies published between 2001 and 2024, we used strict inclusion and exclusion criteria to ensure scientific rigor. Results: The review integrated empirical findings with established theoretical frameworks, particularly from cognitive development and media psychology, to highlight both the advantages and risks of early, frequent exposure to technology. The potential for digital devices to enhance cognitive skills, such as multitasking and information processing, was weighed against risks such as cognitive overload, diminished attention spans, and impaired social skills. We also examined psychological and behavioral outcomes, including identity formation, emotional regulation, and maladaptive behaviors associated with excessive screen time. Additionally, we identified strategies to mitigate negative effects, emphasizing structured digital engagement and parental involvement to support healthy cognitive and psychological growth. Our findings provided actionable recommendations for parents, educators, and policymakers, promoting optimal digital practices that enhanced cognitive development while safeguarding against potential harms. Conclusions: The review offered essential insights for stakeholders in child development, education, and policy-making, highlighting the need for balanced integration of digital tools in childhood learning environments.

## 1. Introduction to Digital Device Usage and Childhood Cognitive Development

In recent years, the rapid integration of digital devices into daily life has significantly reshaped the environments in which children develop, prompting concerns over their impact on cognitive growth [1,2,3,4,5]. While early and frequent exposure to digital devices, such as smartphones, tablets, and computers, has been hailed for offering cognitive enhancements, a growing body of evidence suggests that great exposure may also carry substantial risks [6,7,8]. A 2021 study, for instance, found that children are acquiring their first phones at an average age of 12.2 years, marking a critical shift in the timing and intensity of digital engagement [1,9]. Thus, an accelerating trend necessitates a nuanced exploration of the shift’s positive and negative cognitive ramifications [6,7,10,11].

However, the current body of literature presents conflicting outcomes. While digital devices offer opportunities for cognitive engagement through interactive educational platforms, concerns are emerging over their potential to impair cognitive functions, such as attention, memory, and socio-emotional development, especially when usage is excessive or unregulated [6,12,13]. Such duality—technology as both a means of cognitive enhancement and a potential impediment—complicates our comprehension of its impact. Cognitive development has historically been influenced by various environmental factors, including socio-economic status, education, and parental engagement. The digital age, however, introduces an unprecedented variable—technology—that intersects with these factors in complex and often poorly understood ways [1,2,14]. Research has shown that digital devices can either support cognitive growth or exacerbate developmental challenges depending on the context, content, and duration of use [14,15,16].

The dual-edged nature of technology’s impact is particularly evident when examining its influence on cognitive domains such as attention, memory, executive functions, and social cognition. On the one hand, interactive digital experiences, including video games, have been associated with enhancements in visual-spatial abilities and cognitive flexibility. Educational apps offer promise in fostering problem-solving skills and logical reasoning [14,17]. On the other hand, prolonged screen time has been correlated with attentional deficits, reduced academic performance, and weakened social interaction abilities [12,18]. These conflicting findings emphasize the need for a balanced, context-dependent investigation of how digital devices might foster or hinder cognitive development [19].

Moreover, the relationship between digital device use and cognitive development is not uniform, as it is modulated by individual differences and contextual factors. These include the nature of the content consumed, the interactive versus passive engagement with the device, and the displacement of other developmental activities. For example, the displacement hypothesis posits that screen time might displace time spent on cognitively beneficial activities such as reading and face-to-face interaction, leading to potential developmental delays [2,20]. A comprehensive understanding further substantiates the necessity for a targeted examination of the interaction between digital devices and these diverse developmental contexts [21].

In addition to cognitive implications, digital devices also play a significant role in shaping children’s psychological development. Through social media and other online platforms, children engage in identity formation, emotional understanding, and the development of self-esteem, often with far-reaching implications [22]. For instance, while social media platforms can provide opportunities for social interaction and self-expression, they also present risks related to maladaptive behaviors such as reduced impulse control, social isolation, and addiction-like symptoms tied to excessive device use [23,24]. The psychological ramifications of digital engagement are thus as crucial as the cognitive effects and warrant comprehensive exploration [25].

Given these complexities, our research aims to provide a well-rounded understanding of both the risks and benefits associated with digital device usage in childhood. We recognize the urgency of synthesizing current research to offer practical recommendations for mitigating potential risks while optimizing the benefits [26]. The need for strategic approaches to managing screen time, selecting appropriate content, and balancing digital use with other enriching activities has never been more pressing [27]. Educators, parents, and policymakers play an essential role in ensuring that digital technology supports rather than hinders cognitive and psychological development in children [28].

In conclusion, the relationship between digital device usage and childhood cognitive development is both complex and multi-dimensional. This review aims to provide a comprehensive synthesis of existing literature to highlight where further research is needed, offering valuable insights to stakeholders invested in child development [29]. By understanding the dual potential of digital devices to both enhance and impede cognitive and psychological outcomes, we can better navigate the challenges of raising and educating children in the digital era [30].

### Materials and Methods

The methodology for the above narrative review was organized around an extensive literature search utilizing both primary and secondary sources. We employed an array of scholarly articles, bibliographic indexes, and academic databases, including PubMed, Embase, Scopus, SciELO, Science Direct, and the Web of Science. Our search utilized MeSH-compliant keywords such as “digital device usage”, “childhood cognitive development”, “cognitive abilities”, “executive functions”, and “social cognition”, along with terms reflecting psychological impacts such as “attention span”, “memory”, and “academic performance”. The scope of the literature review was from 2001 to 2024, integrating foundational studies from earlier decades as necessary to provide context and depth to the analysis.

From an initial pool of over 2158 articles, we applied stringent exclusion criteria to refine the collection. These criteria included: (i) relevance to the core topics of digital device impact on cognitive and psychological development in children, excluding studies focused on unrelated medical or psychological conditions; and (ii) exclusion of non-peer-reviewed materials such as Ph.D. dissertations, conference proceedings, and unpublished studies. The above process was crucial to guaranteeing the scientific integrity and pertinence of the incorporated studies.

After applying these criteria, we removed duplicate entries and screened the remaining articles based on their abstracts. This resulted in the selection of 300 articles for full-text assessment. After the final screening phase, 157 articles were included in the review, as they met the high methodological standards required for inclusion. These studies were critically reviewed and discussed among the eight authors, with each subtopic being assigned and decided through consensus. Such a collaborative approach ensured a comprehensive and multidisciplinary analysis of the selected research, providing a robust synthesis of how digital devices influence childhood cognitive and psychological development.

**Figure 1:**
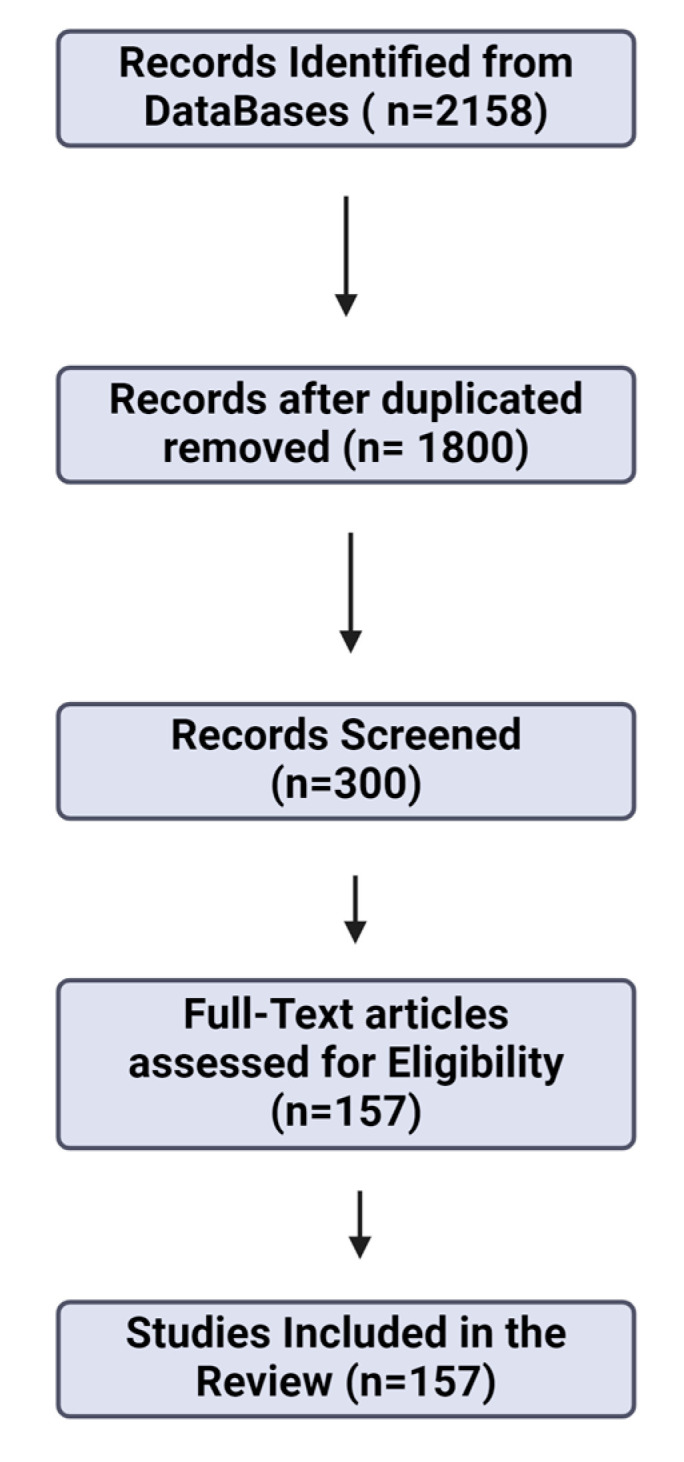
PRISMA flow diagram.

To enhance transparency and provide a clear overview of the screening process, we included a PRISMA flow diagram (see Figure 1). The diagram illustrates the different stages of the review process, from the initial identification of articles to the final selection of studies. The diagram shows the flow of information, including the number of records identified, screened, and excluded, as well as the final studies included in the review. The figure helps to visualize the systematic approach we used in refining the literature for our narrative review, despite its inherent flexibility compared to a systematic review.

Our review protocol was meticulously designed to encompass a wide range of impacts, from enhancing cognitive abilities to potential risks such as reduced attention spans and social skills deficits. By integrating a diverse array of findings, this narrative review aims to provide a balanced perspective on the complex interplay between digital device usage and childhood development, making it a valuable resource for educators, parents, policymakers, and researchers in the field of child development.

## 2. Results

### 2.1. Theoretical Framework: Understanding the Influence of Digital Devices on Cognition

The exploration of how digital devices impact cognitive development necessitates a multifaceted understanding, drawing from both psychological and neurophysiological perspectives [7]. The model delineates the mechanisms by which digital media impacts brain development, cognitive functions, and behavioral outcomes, synthesizing insights from developmental psychology, cognitive neuroscience, and clinical physiology.

#### 2.1.1. Neurophysiological Foundations

From a neurophysiological standpoint, the early and sustained interaction with digital devices significantly influences neural plasticity—the brain’s capacity to reorganize itself by forming new neural connections throughout life. That adaptability is fundamental in how the brain modifies its activities in response to environmental stimuli, which in the context of digital device use includes complex, rapid-paced inputs from screen-based interactions.

Neural plasticity involves several key molecular pathways, prominently featuring the regulation of neurotransmitters and neurotrophic factors. For instance, engaging with digital content requires and stimulates the visual and cognitive systems, leading to an increase in the release of neurotransmitters such as dopamine and glutamate. Dopamine, a neurotransmitter linked to reward and pleasure circuits, plays a pivotal role in learning and attention mechanisms. The frequent and stimulating interactions offered by digital devices can lead to an enhanced release of dopamine, which, over time, might contribute to habit-forming behaviors similar to those observed in addiction [15,27]. Moreover, the increased dopamine activity can affect the brain’s ability to regulate attention and may impair executive functions due to its impact on the prefrontal cortex, a key area involved in decision-making and cognitive control [4,31].

Another crucial aspect is the role of brain-derived neurotrophic factor (BDNF), a protein that supports neuron growth and synaptic plasticity. BDNF levels can be modulated by sensory and cognitive activity, which is abundant during interactions with digital devices. Increased cognitive demands from multitasking and rapid information processing associated with digital media can elevate BDNF expression, thereby influencing neural connectivity and plasticity. Such a mechanism suggests a way that digital device usage could enhance cognitive functions such as visual-spatial skills [5,32].

However, the same mechanisms that potentially enhance certain cognitive abilities can also lead to negative outcomes. Prolonged exposure to screen-based multitasking can overload the brain’s cognitive capacity, leading to a reduction in the efficiency of neural circuits involved in deep, focused thinking and sustained attention. It is aligned with research indicating a reduction in attention span duration and an escalation in task-switching challenges, which are increasingly prevalent with the rise of digital device usage [8]. Furthermore, excessive use of such devices, particularly those requiring rapid response to stimuli, challenges the brain’s executive functions, including cognitive flexibility, working memory, and inhibitory control. These are essential for goal-directed behavior and are crucial in managing new and complex situations efficiently [9]. Thus, the neurophysiological impact of digital devices on cognition encompasses a complex interplay of enhanced sensory and cognitive input, increased neurotransmitter activity, and altered neurotrophic support. These influences can lead to both the enhancement of certain cognitive abilities and the impairment of others, particularly those related to attention and executive function.

#### 2.1.2. Psychological Impact

From a psychological perspective, the impact of digital devices on cognition can be analyzed through the framework of cognitive load theory. It suggests that the capacity of working memory is limited, and an overload of information can lead to decreased learning and retention capabilities. Digital devices, with their capacity to deliver multiple streams of information simultaneously, can often exceed the brain’s processing abilities, particularly in young children whose cognitive systems are still in the developmental stages [10].

The pervasive use of digital devices introduces an environment characterized by continuous partial attention, where a child’s focus is split across multiple tasks simultaneously. Thus, exposure can profoundly influence the development of attentional capacities. In environments dominated by rapid, flashy stimuli from screens, children may find it increasingly challenging to engage in sustained attention or to develop deep processing capabilities, which are crucial for the understanding and integration of complex cognitive tasks [13,16]. Furthermore, digital devices affect the development and operation of executive functions, which are higher-level cognitive processes that regulate and manage other cognitive processes. These functions include skills such as problem-solving, memory, and cognitive flexibility, which are essential not only for academic success but also for daily decision-making and problem-solving. The impact of digital devices on these functions has been shown to be dualistic. While certain digital activities such as educational games and problem-solving apps can enhance these abilities, excessive or misdirected use of digital devices can impair them (Figure 1). For instance, habitual engagement with fast-paced, multitask-oriented games may improve response times and cognitive flexibility, but at the cost of diminished capacity for focused, sustained attention and deeper cognitive processing [11].

**Figure 2:**
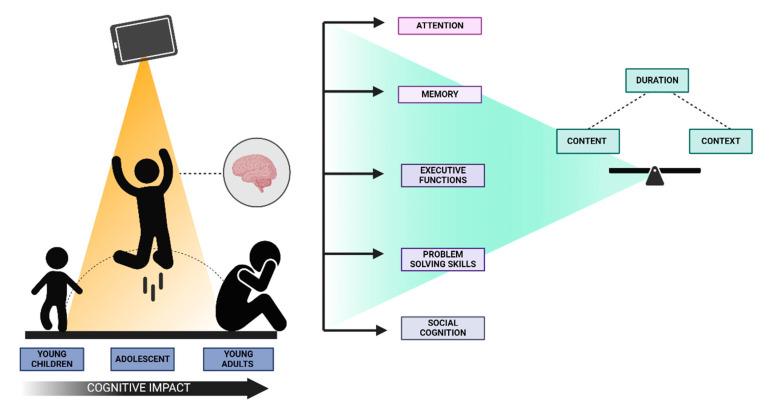
The influence of using digital devices on the development of children and the importance of considering the context, content, and duration of this usage.

The psychological impact is also reflected in the way digital devices shape cognitive development through habitual use patterns. When devices are used excessively for entertainment, such as watching videos or playing non-educational games, they can contribute to cognitive overload, which hampers the development of memory retention and learning processes. Thus, cognitive overload is particularly detrimental during the early developmental stages, where foundational cognitive and learning skills are being established (Figure 2).

Moreover, the interaction between digital device usage and cognitive development is influenced by the nature of the digital content and the context in which devices are used. Educational content designed to be interactive and that which promotes critical thinking and problem-solving can support cognitive development. In contrast, passive consumption of content, such as prolonged video watching or engagement with overly stimulating apps, does not contribute similarly and may even detract from cognitive capacity and executive function development. Thus, while digital devices are integral to modern learning and development, their impact on cognitive processes is complex and necessitates careful management to optimize cognitive development outcomes. Balancing the content, context, and duration of device usage is essential to harnessing the benefits of technology while mitigating the risks associated with cognitive overload and the potential impairment of essential cognitive functions.

#### 2.1.3. Developmental Considerations

Developmental psychology provides crucial insights into the timing and context of digital device exposure and how these factors can influence cognitive outcomes in children. According to Jean Piaget’s theory of cognitive development, active, hands-on experiences are essential for cognitive growth at various developmental stages. Piaget posited that children progress through a series of stages of cognitive development, each characterized by different abilities and ways of thinking. The sensorimotor stage (birth to about 2 years) and the preoperational stage (ages 2 to 7) emphasize physical interaction with the environment and symbolic play, respectively. These stages are critical for the development of sensorimotor skills, logical thinking, and problem-solving abilities [17,18].

Digital devices, while beneficial in certain contexts, can sometimes replace these essential hands-on learning opportunities. When screen time supplants activities such as physical play, interactive learning, and face-to-face social interactions, it may impede the development of critical cognitive skills. For instance, research has shown that excessive screen time is associated with delays in language development, which is particularly pronounced in young children who need verbal interactions for language acquisition [19]. Furthermore, a study by Huber et al. (2018) found that children who engaged more frequently with digital devices showed lower levels of executive function, including poorer working memory and inhibitory control, compared to those who engaged in more traditional play activities [20].

Lev Vygotsky’s socio-cultural theory of cognitive development underscores the importance of social interactions in the developmental process. Vygotsky argued that cognitive development is largely driven by social interactions and that learning is fundamentally a social process. According to his theory, children learn through guided participation and collaborative dialogues with more knowledgeable others, such as parents, teachers, and peers [21]. Digital devices, while offering new avenues for social interaction through social media and online games, can also reduce face-to-face interactions. Such reduction can hinder the development of social cognition, empathy, and emotional regulation skills, which are fostered through direct human contact and socialization.

The potential negative impact of digital devices on social development is supported by empirical research. For example, a study by Twenge and Campbell (2018) reported that increased screen time was associated with lower psychological well-being, including higher levels of loneliness and depression among adolescents [23]. These findings suggest that while digital interactions can supplement traditional social interactions, they cannot fully replace the benefits of face-to-face communication. Additionally, a longitudinal study by Przybylski and Weinstein (2017) indicated that moderate digital device use was not harmful and could be beneficial, but excessive use was correlated with decreased well-being and impaired social skills [22].

The context in which digital devices are used also plays a crucial role in their impact on cognitive development. Structured and educational use of digital devices can support learning and cognitive development. For example, interactive educational apps designed to promote literacy and numeracy skills can be effective tools for learning [15]. However, unstructured and passive use, such as watching videos without any interactive component, is less likely to be beneficial and may even be detrimental. A meta-analysis found that the effectiveness of digital learning tools is significantly influenced by how they are integrated into the learning environment, with more interactive and guided uses being more effective [24,25]. Moreover, the timing of digital device exposure is critical. Early childhood is a period of rapid brain development, and excessive screen time during these formative years can have lasting impacts. The American Academy of Pediatrics (AAP) recommends that children younger than 18 months avoid the use of screen media other than video chatting and that children aged 2 to 5 years should be limited to one hour per day of high-quality programming. These guidelines emphasize the need for parental involvement in ensuring that digital device use is appropriate and beneficial.

Thus, developmental considerations highlight the importance of balancing digital device use with traditional hands-on learning experiences and face-to-face social interactions. While digital devices can offer valuable educational opportunities, they should not replace the critical activities that support cognitive and social development. Ensuring that digital device use is structured, interactive, and limited in duration can help maximize the benefits while mitigating the potential risks. Thus, a balanced approach is essential for fostering healthy cognitive and social development in children.

### 2.2. Enhancing Cognitive Abilities: Positive Effects of Digital Device Usage

The increasing integration of digital devices into everyday life has sparked significant interest in understanding their effects on various aspects of childhood cognitive development. Contrary to the often-highlighted negative impacts, a growing body of research suggests that digital devices can also enhance certain cognitive abilities in children. That perspective invites a nuanced examination of how digital devices, when used appropriately, can serve as powerful tools for cognitive development.

Digital devices, including tablets, smartphones, and computers, offer interactive and engaging experiences that can foster cognitive skills. One of the primary positive effects noted in the literature is the enhancement of visuospatial skills. Studies have shown that video games, particularly those requiring spatial navigation, hand-eye coordination, and rapid processing of visual information, can significantly improve visuospatial abilities in children [5]. These skills are crucial for success in various academic and everyday tasks, such as reading maps, understanding graphs, and navigating physical spaces. Indeed, video games, especially those in the action and adventure genres, often require players to manipulate objects and navigate through complex three-dimensional environments. Consequently, interaction necessitates the constant adjustment of spatial orientation and coordination, thereby honing visuospatial skills. Research by Spence and Feng [24] demonstrates that action video games can enhance a player’s ability to track multiple objects simultaneously, a skill that is directly transferable to real-world tasks such as driving and performing surgery [24]. The ability to visualize and manipulate objects in space is also fundamental in STEM (Science, Technology, Engineering, and Mathematics) education. For instance, students who regularly engage with spatially demanding video games perform better in subjects such as geometry and physics [25].

Moreover, the rapid advancement of augmented reality (AR) and virtual reality (VR) technologies has introduced new dimensions to the potential cognitive benefits of digital devices. AR and VR applications immerse users in environments that require active participation and manipulation of virtual objects. These technologies are increasingly used in educational settings to teach complex concepts in an engaging manner. A study by Cheng and Tsai [26] found that VR-based learning environments significantly enhanced students’ spatial understanding and scientific reasoning [26]. Concretely, an immersive learning experience can make abstract concepts more tangible and easier to grasp, thereby improving cognitive skills related to spatial awareness and problem-solving. In addition to these advanced technologies, simpler digital tools and apps designed for younger children can also foster visuospatial development. For example, drawing and puzzle apps encourage children to recognize patterns, shapes, and spatial relationships. These activities support the development of early mathematical skills, such as geometry and measurement, which are foundational for later academic achievement [27]. Furthermore, interactive storybooks and educational games often include elements that require children to follow directions, recognize spatial relationships, and predict outcomes based on spatial information, further enhancing their visuospatial abilities [28].

#### 2.2.1. Visuospatial Skills

Enhancing visuospatial skills through digital device usage can benefit children with specific learning disabilities, such as dyslexia, which often involves difficulties with spatial orientation and visual processing. Interventions that include targeted video game play or AR activities can provide these children with enjoyable and effective ways to improve their visuospatial skills [29]. Additionally, children with developmental disorders such as autism spectrum disorder (ASD) may benefit from digital tools designed to enhance visuospatial skills, as these tools can provide structured environments for practicing navigation and spatial awareness, which are areas often challenging for individuals with ASD [30]. Moreover, the positive effects of digital device usage on visuospatial skills extend to physical health and rehabilitation. For example, VR has been successfully used in therapeutic settings to improve motor coordination and spatial awareness in children with cerebral palsy. These therapies leverage the engaging nature of digital environments to motivate patients and provide real-time feedback, which is crucial for effective rehabilitation [33].

#### 2.2.2. Language Acquisition and Literacy Development

Another area where digital devices have demonstrated positive effects is in language acquisition and literacy development. Educational apps and e-books are designed to make learning interactive and fun, often incorporating multimedia elements that cater to different learning styles. For instance, a study by Neumann and Neumann [31] found that young children who used tablet-based e-books showed improved early literacy skills compared to those who used traditional print books [31]. The interactive features of digital books, such as animations and embedded games, can enhance engagement and motivation, leading to better learning outcomes. Additionally, these digital tools often include features such as word highlighting and audio narration, which can aid in vocabulary development and phonemic awareness [34]. Moreover, the adaptive learning technologies embedded in many educational apps provide personalized feedback and adjust the difficulty level according to the child’s progress, further supporting individualized learning [34]. Thus, digital devices can be potent tools for enhancing early literacy and language skills.

#### 2.2.3. Cognitive Development

Digital devices can support cognitive development through educational games that promote problem-solving and critical thinking. These games often require children to follow complex instructions, make strategic decisions, and solve puzzles, which can enhance their cognitive flexibility and executive functioning [35]. For example, games that involve building structures or managing resources, such as “Minecraft” and “SimCity”, can teach children how to plan, prioritize tasks, and think logically. These games often require players to allocate resources efficiently, plan long-term strategies, and adapt to changing scenarios, thereby honing their executive functioning skills. A meta-analysis by Granic et al. [36] highlighted that video games could improve a range of cognitive skills, including attention, spatial skills, and problem-solving abilities [36]. Games such as “Portal” and “The Legend of Zelda” series require players to solve complex puzzles and navigate intricate environments, which can significantly enhance their problem-solving skills and cognitive flexibility. These games challenge players to think critically and creatively to progress through various levels, fostering an environment that promotes cognitive growth. Additionally, educational apps such as “DragonBox” and “Math Blaster” make learning math concepts engaging and interactive. These apps often present mathematical problems in the form of games, requiring children to apply critical thinking and problem-solving skills to achieve high scores or advance to higher levels. By integrating educational content with game mechanics, these apps can make learning more enjoyable and effective, leading to better retention and understanding of complex concepts.

The benefits of these digital tools extend to collaborative and competitive environments as well. Multiplayer games such as “World of Warcraft” and “Among Us” encourage teamwork and strategic thinking. Players must communicate, collaborate, and develop strategies to achieve common goals or outsmart opponents, which can improve social cognition and cooperative problem-solving skills [37]. These social interactions within the gaming environment can also help develop communication skills and the ability to work effectively in a team. Indeed, research has shown that these cognitive benefits are not limited to entertainment games. Serious games, designed explicitly for educational purposes, also demonstrate significant positive impacts on cognitive development. For example, “Foldit”, a puzzle game about protein folding, has been used in scientific research to crowdsource complex scientific problems. Players’ contributions have led to real-world scientific discoveries, demonstrating the potential of games to engage users in complex problem-solving tasks with meaningful outcomes [38].

#### 2.2.4. Memory Processes, Creativity, Social Cognition, and Collaborative Learning

Attention, another critical cognitive domain, can also benefit from the use of digital devices. While excessive screen time is often linked to reduced attention spans, controlled and purposeful use of digital media can have the opposite effect. Interactive tasks that require sustained attention and quick responses can help improve children’s attentional control and selective attention [26]. A study by Dye et al. found that children who regularly played action video games exhibited faster reaction times and greater attentional capacity than those who did not play these games [39]. Then, it is suggested that not all digital devices use negatively impacts attention, and certain types of digital interactions can enhance this cognitive skill. Furthermore, specific genres of video games, such as first-person shooters and real-time strategy games, demand high levels of concentration and rapid information processing. These games require players to monitor multiple stimuli simultaneously, make quick decisions, and respond to changes in the environment promptly. For example, games such as “Call of Duty” and “StarCraft” necessitate sustained attention and strategic planning, which can translate to improved attentional control in real-world scenarios [40].

In educational contexts, digital tools such as interactive learning apps and online modules are designed to maintain children’s engagement through gamified learning experiences. Programs such as “Khan Academy Kids” and “ABCmouse” utilize adaptive learning techniques to keep students focused on tasks that are appropriately challenging, thus enhancing their ability to sustain attention over extended periods [29]. These tools provide immediate feedback and rewards, which can motivate students to stay attentive and improve their focus. Additionally, mindfulness and attention-training apps, such as “Headspace for Kids” and “Breathe, Think, Do with Sesame”, are designed to help children develop better attention regulation and mindfulness skills. These apps guide children through exercises that improve their ability to concentrate and manage distractions, contributing to better overall attentional control [37]. By incorporating regular use of these apps into daily routines, children can practice and enhance their attentional skills in a structured manner. Research also indicates that digital learning environments can help children with attention-deficit/hyperactivity disorder (ADHD) manage their symptoms more effectively. For instance, programs such as “EndeavorRx”, a therapeutic video game approved by the FDA, are specifically designed to target attention control and cognitive functioning in children with ADHD. Studies have shown that regular use of such programs can lead to measurable improvements in attentional performance and overall cognitive function in affected children [41].

Educational apps and games that require children to remember sequences, patterns, or pieces of information can strengthen both short-term and long-term memory. For instance, memory-matching games, commonly found in educational apps, require players to remember the locations of different items and can improve working memory capacity [42]. Additionally, the multimedia elements of digital learning tools, such as videos and interactive simulations, can aid in better information retention by providing multiple modes of representation and engaging different cognitive pathways [43]. Also, creativity and problem-solving skills are other areas where digital devices can offer substantial benefits. Digital art tools, music composition apps, and game design platforms allow children to express their creativity in novel ways. Such tools often provide immediate feedback and endless opportunities for experimentation, which can foster creative thinking and innovation [44]. For example, a study by Jackson et al. found that children who engaged in digital storytelling activities showed significant improvements in creative writing skills and overall creativity [45]. The interactive and iterative nature of these digital tools encourages children to explore, experiment, and refine their ideas, thereby enhancing their creative capacities.

Furthermore, digital devices can facilitate social cognition and collaborative learning. Many educational platforms and games are designed to be used in group settings or online communities, promoting social interaction and cooperative problem-solving. These activities can help children develop important social skills, such as communication, empathy, and teamwork [37]. For example, multiplayer online games often require players to work together to achieve common goals, which can enhance their ability to cooperate and collaborate with others. A study by Vygotsky [21] emphasized the importance of social interaction in cognitive development, and digital devices can provide new avenues for such interactions [21]. In addition to these cognitive benefits, digital devices can support personalized learning experiences tailored to individual needs and preferences. Adaptive learning technologies use algorithms to adjust the difficulty level of tasks based on the user’s performance, ensuring that each child is challenged at an appropriate level. A personalized approach can lead to more effective learning and better cognitive outcomes [32]. For instance, mathematics apps that adapt to the learner’s skill level can help children master concepts at their own pace, reducing frustration and increasing motivation to learn.

While the potential benefits of digital device usage are significant, it is crucial to approach their integration into children’s lives with careful consideration and balance. Excessive or inappropriate use can lead to negative outcomes, such as reduced physical activity, sleep disturbances, and an increased risk of digital addiction [46]. The key to harnessing these benefits lies in balanced usage, appropriate content selection, and active involvement of caregivers and educators in guiding children’s digital experiences. As digital technologies continue to evolve, ongoing research and adaptive strategies will be necessary to ensure that they contribute positively to the cognitive development of future generations. Therefore, it is essential for parents, educators, and policymakers to establish guidelines and practices that maximize the positive effects of digital devices while mitigating potential risks.

### 2.3. Attention: Potential Enhancements and Detriments on Attention Span and Focus

Extensive research in cognitive psychology has shown that humans have a limited ability to sustain attention and can only concentrate on a particular task or stimulus for a restricted duration. That ability is not constant and changes based on various factors, including the nature of the task and individual influences such as interest, motivation, and personal experience [47,48].

Attention span and focus are critical components of cognitive functioning that impact various aspects of daily life, including academic performance, workplace efficiency, and overall mental health. The pervasive integration of digital devices in daily life has sparked considerable interest in understanding their effects on childhood cognitive development. Attention span and focus, critical components of cognitive development, are believed to be significantly influenced by early and frequent interaction with digital technologies. This review aims to consolidate recent scientific findings to provide a comprehensive understanding of these effects.

Several studies have identified potential cognitive benefits associated with the use of digital devices, particularly educational applications, and interactive learning tools. One of the enhancements is the improved learning and educational outcomes. In this way, some research found that interactive educational apps can enhance learning experiences by providing immediate feedback and personalized learning paths, thus improving cognitive functions such as problem-solving and critical thinking [21,49]. Others demonstrated that digital games designed for educational purposes can improve memory retention and increase engagement in learning activities [50].

In the same way, it should be noted the enhanced in multitasking abilities. Rosen et al. reported that children who regularly use digital devices may develop better multitasking skills, which could be attributed to the rapid switching between tasks required when using these devices [51]. Other authors suggested that moderate use of digital devices might enhance the ability to process information from multiple sources simultaneously, thereby improving cognitive flexibility [52].

On the other hand, excessive or inappropriate use of digital devices has been linked to several negative outcomes, particularly concerning attention span and focus. It reduced attention span; Muennig et al. highlighted that prolonged screen time, especially on fast-paced and highly stimulating content, can lead to a reduction in sustained attention and an increase in attention-related problems in children [53]. Other authors found that children exposed to digital devices for more than two hours per day exhibited shorter attention spans and higher levels of distractibility compared to those with limited screen time [54]. Twenge and Campbell discussed the negative correlation between heavy digital device usage and academic performance, connecting those variables to decreased focus and increased instances of digital distraction [55]. In this way, Radesky et al. reported that the constant interruptions from notifications and the immersive nature of digital content can significantly impair a child’s ability to concentrate on tasks, both academic and non-academic [56].

In fact, other negative effects that can be seen include sleep disruptions, which described LeBourgeois et al., who indicated that screen exposure, particularly before bedtime, can disrupt sleep patterns, which in turn negatively impacts cognitive functions, including attention and memory [57]. Other research found that poor sleep quality linked to late-night digital device usage can lead to daytime sleepiness and decreased cognitive performance [58]. Several studies emphasize the role of moderating factors such as the type of content, duration of use, and parental involvement. Anderson and Subrahmanyam stressed that not all digital content is detrimental; educational and age-appropriate content can have positive cognitive effects [59]. Straker et al. argued that the quality of digital content plays a crucial role in determining its impact on cognitive development [60]. Lauricella et al. highlighted the importance of parental guidance in mitigating the negative effects of digital device usage by setting limits and ensuring children engage with high-quality content [61]. Hutton et al. found that co-viewing and discussing digital content with children can enhance learning outcomes and mitigate attentional issues [28].

The impact of digital device usage on childhood cognitive development is multifaceted, with both potential enhancements and detriments observed. The key to optimizing these effects lies in moderating the amount and type of digital content children are exposed to, coupled with active parental involvement. Future research should continue to explore these dynamics to provide clearer guidelines for integrating digital devices into children’s lives in a way that supports healthy cognitive development.

### 2.4. Memory: Positive and Negative Impact of Digital Devices on Cognitive Processes

The proliferation of digital devices has dramatically changed how individuals interact with information and perform cognitive tasks. Modern children’s fascination with television and smart electronic devices reduces the time they spend communicating and playing, which inevitably affects their developmental outcomes. Children’s use of media and technology starts at an early age, with television remaining the most popular medium among them [62]. However, children also actively use smart electronic devices such as tablets and smartphones. It is clear that these types of screen time engage children differently and have varying impacts on their development [63].

#### 2.4.1. Positive Impacts of Digital Devices on Memory and Cognitive Processes

Recent studies have shown that certain cognitive enhancement apps can have a positive impact on memory and other cognitive functions. Apps designed to improve working memory, attention, and problem-solving skills have been found to produce beneficial effects. For instance, a recent study demonstrated that regular use of brain-training apps can lead to improvements in working memory and fluid intelligence, especially in older adults [64].

Digital notetaking, particularly with the use of stylus and tablets, has been found to enhance memory retention and comprehension. Research by Morehead, Dunlosky, and Rawson indicates that taking notes on digital devices can be as effective as traditional note-taking methods, provided that active learning strategies are employed. The ability to organize, search, and retrieve notes easily also adds to the advantages of digital note-taking [65].

Digital devices provide access to a vast amount of information and educational resources, enabling personalized learning experiences that can enhance memory and cognitive processes. A study by Mayer highlights that multimedia learning, which combines text, audio, and video, can improve memory retention by catering to different learning styles and providing richer contextual cues [66].

#### 2.4.2. Negative Impacts of Digital Devices on Memory and Cognitive Processes

One of the most significant negative impacts of digital devices is related to digital media multitasking. Numerous studies have shown that multitasking with digital devices can impair working memory and cognitive control. For instance, Uncapher and Wagner found that heavy media multitaskers perform worse on memory tasks and exhibit reduced ability to filter out irrelevant information, which can lead to decreased cognitive performance over time [67].

The constant influx of information from digital devices can lead to cognitive overload, making it difficult to process and retain information effectively. It has been discussed how smartphones can reduce available cognitive capacity, as individuals are often preoccupied with potential notifications and distractions, thus impairing memory and attentional control [68].

Digital devices often promote shallow processing of information due to the ease of access and rapid consumption of content. Research by Carr indicates that the habit of skimming through digital content, rather than engaging in deep reading, can hinder memory consolidation and the ability to form long-term memories. That shift from deep learning to surface-level engagement can have detrimental effects on overall cognitive development [69].

An excessive reliance on digital devices for information retrieval can negatively impact memory retention. Sparrow, Liu, and Wegner describe the “Google effect”, where individuals are less likely to remember information that they know they can easily look up online. This externalization of memory reduces the need for internal cognitive processing, potentially weakening memory skills over time [70].

The impact of digital devices on memory and cognitive processes is multifaceted, with both positive and negative outcomes. While cognitive enhancement apps and digital notetaking can support memory and learning, digital media multitasking, information overload, and dependence on devices pose significant challenges. Future research should continue to explore strategies to maximize the cognitive benefits of digital technology while mitigating its potential drawbacks. Understanding the nuanced effects of digital devices on cognitive processes will be crucial for developing effective educational tools and promoting healthy cognitive habits in the digital age.

### 2.5. Executive Functions: Exploring Digital Device Usage and Cognitive Control and Decision Making

Executive functions are defined in the literature as the set of cognitive skills that allow people to plan, make decisions, and solve problems. In short, these skills allow directing behaviors towards an objective or goal in an effective way. Although the scientific literature includes different components of executive functions, the most studied of them all is undoubtedly inhibitory control, which is crucial in decision-making [71]. The executive functions have a significant role in human learning, leading to much research in this area, particularly in the field of education and children with special educational needs and disabilities [72].

In this regard, the use of digital devices is emerging as a form of evaluation and training of executive functions no longer only in the clinical field, if not in the field of formal and non-formal education, highlighting the increasing importance of uniting the knowledge derived from neuroeducation, technology, and the educational system [73]. Through their research, they showcased the pivotal role of inhibitory control in regulating other executive processes and highlighted the potential for enhancing these abilities through computer programs [74]. Another relevant study in the field is that developed by Ramos and García (2019) in the Specialized Educational Service to evaluate the use of digital games in children previously identified with difficulties in the performance of inhibitory control [75]. Their results go in line with the previous ones, highlighting the use of digital games and devices in the training of cognitive functions and cognitive control in particular, showing significant improvements in this sample. However, these results have not only been observed in the youth population, but in the study conducted by Najberg et al. (2021), with older adults the results go in the same direction [76].

Various authors have concluded that the use of digital devices enhances the development of executive functions, such as cognitive control and decision-making. These findings hold true across different samples. However, caution should be exercised in interpreting these results, considering the findings of Russo-Johnson et al.’s (2017) research, which revealed the influence of other variables, such as age, gender, and socio-economic level, on the expected outcomes [72,77].

Therefore, it can be concluded that, although there seems to be some consensus among the authors in stating that inhibitory control and decision-making training using digital devices is widespread and promising, other variables should be considered when designing these devices so that interventions are as effective as possible.

### 2.6. Creativity and Problem-Solving Skills: Opportunities and Challenges

The integration of digital technology into daily life, especially the lives of children, is steadily growing. Portable digital gadgets are ubiquitous and have become integrated into activities that previously did not necessitate their use. The abilities that are connected to this topic are commonly known as 21st-century skills, which encompass a novel form of literacy known as digital literacy [78]. In this regard, creativity is the initial spark that ignites every achievement, achieved by altering the typical cognitive framework of information [79]. The absence of creativity would have hindered the progress of human evolution. Creativity is a skill that may be enhanced or influenced over time by one’s surroundings and the activities they engage in. To boost creativity, several strategies can be employed in different settings and by diverse user groups [80].

The school setting, including all levels of education, including homeschooling or e-schooling, is the most appropriate place for the utilization of visual mnemonic devices [81]. These technologies and talents introduce distinctive and inventive aspects to the learning process; nonetheless, the impact of such experience on behavior, emotion, and socialization remains uncertain. Interacting with digital devices and educational games can cause bewilderment and boredom in preschool-aged children. These negative emotions might lead to mind-wandering and exploration, which can actually help facilitate learning [82].

The utilization of digital media has been documented to provide both adverse and beneficial consequences. Concerning the first point, excessive media consumption has been linked to impaired motor skills and higher levels of physical inactivity [83] and other physical health effects [7]. It has also been associated with reduced attention, negative psychological effects on mental health, cognitive abilities, and academic performance, as well as hindered language development [84]. These findings may be attributed to a limited amount of interaction between children and their parents, as well as a decrease in the amount and quality of playtime for these children [85]. This is particularly important because research has shown that a child’s indirect interactions with an adult caregiver, such as receiving praise, following indirect commands, and answering questions from the caregiver, can enhance their self-esteem, curiosity, and ability to think creatively [86]. In this regard, there was no correlation observed between the level of creativity and the duration of computer game play among preschool-aged children. However, Bukhalenkova et al.’s investigation uncovered substantial correlations between creativity and the attributes of parental involvement in the utilization of gadgets by preschoolers. That study demonstrated that children who engage in collaborative play with siblings or peers while using electronic devices exhibit considerably higher scores in imaginative flexibility compared to those who frequently engage in solitary play or play with adults [87].

#### 2.6.1. Mnemonic Devices

The term “mnemonic” pertains to memory or is associated with memory. Mnemonic devices are cognitive strategies that can be employed to encode information, resulting in enhanced retention of the concepts provided [88]. The devices involve verbally listing, categorizing, or defining one or more concepts, as well as the mental process of forming visual representations of the items involved. Cioca et al. reported that visual mnemonic devices had a beneficial effect on creativity. However, there are no empirical studies that demonstrate the correlation between visual mnemonic devices and creativity in terms of their influence on creative performance. Conversely, certain research discusses the inverse correlation, namely the influence of creativity on visual mnemonic performance. The effectiveness of visual imagery and mnemonics in [89] relies on an individual’s originality. Therefore, a higher creativity score has the potential to enhance performance when using visual mnemonic devices [90]. Additionally, research has demonstrated that the utilization of mnemonics helps augment pupils’ capacity to efficiently arrange and recall knowledge. Consequently, this aids in enhancing the pupils’ self-esteem and their acquisition of knowledge [91]. Additionally, visual mnemonics can be utilized to connect words with their meanings [92]. For instance, the use of creative-map mnemonic procedures can be inferred to have a beneficial effect on the acquisition and retention of geographical place names and places [93]. Also, Hill and collaborators specified that mnemonic devices can effectively enhance a student’s ability to remember terminology [94]. Concretely, Chinese students learning English as a foreign language show a preference for mnemonic strategies that help them bridge the linguistic differences between their native language and the target language [95]. Subsequently, we will explore the impact of mnemonic devices on teaching and learning processes, as well as the role of artificial intelligence and smart personal assistants in enhancing the creative process of education for both students and teachers.

#### 2.6.2. Smart Personal Assistants and AI

Smart personal assistants (SPAs), such as Amazon’s Alexa or Google’s Assistant, enable users to engage with computers in a manner that is both more intuitive and advanced than previously conceivable [96]. Despite the growing body of research on SPA technology in education, there is a lack of empirical evidence about its effectiveness in providing dynamic scaffolding to improve students’ problem-solving skills. However, evidence showed that groups exhibited significant improvements in their problem-solving abilities, and we saw notable shifts in their learning approaches. Through the utilization of Single Page Applications (SPAs), students were able to cultivate personalized relationships and obtain tailored assistance on their everyday devices [97]. When compared to groups working with human tutors, smart personal assistant tutors exhibit noticeably better task outputs and higher levels of collaboration quality. The findings are utilized to propose potential avenues for further investigation in the domain of computer-supported collaboration [98]. In addition, enabling educators to create smart personal assistants that greatly enhance students’ academic achievements [99]. However, a recent study pointed out that teams facing time scarcity relied on the intelligent assistant more frequently and demonstrated lower performance on a creative job in comparison to teams without time constraints. In addition, teams that had access to an intelligent assistant experienced reduced member interactions compared to teams without the technology [100]. Therefore, it is advisable to proceed with caution when utilizing these technologies. Artificial creativity scholars contend that users of (Artificial Intelligence) AI systems must comprehend the programming of the conceptual space and the limitations under which computational systems exhibit creativity instead of attributing human-like qualities or idolizing machine creativity. They emphasize the importance of discussing, critiquing, exploring, and experimenting with these aspects to fully engage with AI systems [101,102]. Generally, it has been recognized the immense capacity of AI to enhance, amplify, and transform human creativity [103,104]. The understanding is that the combination of creativity with AI has the potential to generate a range of different futures, each possessing distinct qualities and possibilities. The organization is dedicated to creating a supportive atmosphere that values and promotes innovative self-expression while also ensuring the responsible and ethical use of AI. The objective is to establish a global environment where humans and artificial intelligence coexist harmonically, collaborating to fully optimize the potential of creativity across all domains of life [103]. Focusing on students’ opinions, those who are indicated to have a greater level of self-perceived comprehension of AI expressed more favorable attitudes toward incorporating AI into their educational environments. However, students with limited comprehension of AI tended to experience apprehension towards AI. The majority of pupils demonstrated a comprehensive comprehension of creativity and expressed that artificial intelligence could never attain the same level of creativity as humans [105]. It is crucial to acknowledge the obstacles and disruptions that arise from the usage of AI and to recognize the issues that educators may encounter when incorporating these technologies into learning environments. This entails evaluating the concept of AI as a necessity in education and advocating for educators to adopt a discerning approach to address the apparent advantages of AI systems [106].

### 2.7. Social Cognition: Effects of Digital Devices on Social Interaction

The emergence of the Internet has represented a great advance in the development of societies, which is increased today with the appearance of new digital devices that are incorporated in the day-to-day of people [107]. This incorporation implies changes both in the social organization and in such basic elements of the human being as social cognition. Beaudoin and Beauchamp (2020) define social cognition as the set of mental abilities acquired throughout life that allow individuals to perform other cognitive activities such as perception, processing, and interpretation of social-cutting stimuli, thus facilitating social responses and adaptation. Therefore, the importance and relevance of the study of the effect of these devices on social interaction cannot be denied [108].

There seems to be some consensus in the literature that the effect of these digital devices on development and social interaction will depend to a large extent on different factors, including the age of the children, parental involvement, and finally the content worked or consumed through these devices. With regard to the age of minors, various studies have highlighted the negative effects of these devices on the development of juveniles, mainly those relating to the consumption of visual content. It has been observed, for example, that infants and preschool children who use these devices subsequently present greater cognitive and language retardation aspects that will affect their subsequent social interaction. The main reason for these observed disadvantages lies in unmediated exposure by parents and by the type of content [109,110]. Since contrary results have been found from the use of these devices in these children as long as there is an appropriate parent-child interaction and the content is pro-social [111].

We must not forget that in this period the brain of the children is exposed to a great change and maturity, and the use of these devices will have a direct effect on this development. In this respect, it has been possible to observe how the excessive use of these devices is related to a reduction in the melanization of the white substance (Hutton et al., 2020). Therefore, many associations, such as, for example, the Spanish Association of Pediatrics in Primary Care (2018), do not recommend the use of this type of screen and device in children under 18 months [112].

With regard to adolescence, the data provided in the literature are controversial [113]. Some authors argue that excessive use of these devices increases the likelihood of suffering from psychosocial, emotional, behavioral, and social difficulties, resulting in social interaction problems [114]. Many authors and specialists are even concerned about the phubbing, a phenomenon caused by the excessive use of smartphones and social networks that is increasingly being seen in the younger population with a direct impact on their interpersonal interaction capabilities [115].

However, it should also be commented that there are other authors who defend that the use of, for example, games on the network favors socialization as they stimulate basic skills in this process, such as empathic development [7]. In addition, these benefits of social interaction have also been observed in the educational context, as the use of mobile in classrooms, a phenomenon known as mobile learning, also stands out for opportunities to generate more collaborative learning among students, an aspect highlighted by themselves and by the teachers themselves [116].

### 2.8. Mitigating Risks: Strategies for Balancing Digital Device Usage

In the contemporary era characterized by rapid technological progress, digital devices have become essential components of our everyday lives [117]. These tools offer numerous advantages, including enhanced communication and improved educational and professional experiences. However, the excessive or unbalanced use of digital devices can also negatively impact individuals, particularly affecting students’ social interactions and mental health [118].

In this line, recent studies have underscored the complex relationship between digital device usage and various aspects of well-being [119]. Although social media can enhance connectedness and access to information, excessive use can hinder face-to-face communication and contribute to mental health problems [120]. Thus, Silva et al. [121] reported that disproportionate use of digital devices can disrupt in-person social interactions and the development of relationships among medical students. Similarly, Gupta et al. [122] founded that the overuse of social media can lead to issues such as eye strain, fatigue, physical inactivity, reduced attention span, and sleep disorders, along with decreased self-esteem, eating disorders, anxiety, and feelings of inferiority.

These findings highlight the importance of developing strategies to maintain a healthy balance between digital device use and other vital aspects of life [119]. Implementing measures to limit smartphone and social media use, particularly before bedtime, can mitigate adverse effects on mental health and academic performance [123]. Additionally, encouraging students to engage in regular physical activities, practice mindfulness, and prioritize face-to-face social interactions can promote a more balanced and fulfilling lifestyle [124].

Researchers have explored various approaches to address the challenges associated with digital device usage. For instance, a study on university students found that reducing digital media use two hours before bedtime can positively affect sleep quality, subsequently supporting physical and mental well-being [125]. Moreover, the “always-on” culture fostered by digital technologies has created unreasonable expectations of constant availability and rapid responses, which can negatively impact mental health. As digital devices have become pervasive in modern life, it is crucial for individuals, especially students, to develop strategies to balance their use and maintain a healthy lifestyle [126].

One effective strategy is to establish clear boundaries and prioritize face-to-face interactions. While technology provides valuable opportunities, recognizing the importance of in-person social interactions and real-world relationship building is essential. Students should allocate specific times for digital activities and consciously engage in physical activities, social gatherings, and other non-digital hobbies to maintain a healthy balance [127]. Another crucial strategy is to be mindful of the impact of digital device usage on sleep patterns and overall well-being. According to this, studies have shown that exposure to digital devices, particularly at night, can adversely affect sleep quality, which in turn can influence cognitive function, mood, and physical health [128]. Therefore, students should establish a consistent sleep routine, limit digital device use before bedtime, and prioritize quality sleep to maintain optimal well-being [128].

In conclusion, balancing digital device usage is a critical challenge for individuals, especially students, in the modern era. By implementing strategies such as prioritizing in-person interactions, managing sleep patterns, and developing self-awareness, individuals can maintain a healthy and balanced relationship with digital devices, ensuring they reap the benefits while mitigating potential negative consequences.

### 2.9. Digital Device and Academic Performance

In the rapidly changing educational landscape, digital devices have become increasingly prominent, stimulating critical discussions regarding their impact on academic performance [129]. The extensive adoption of technologies, such as laptops, tablets, and smartphones, has transformed the learning environment, creating both opportunities and challenges for students and educators.

Numerous studies have investigated the relationship between the academic use of mobile technology and higher order thinking skills. The findings indicate that the academic utilization of mobile technology can significantly influence students’ higher-order thinking abilities, learning effort, and active course engagement [130]. Thus, it is particularly relevant for the “digital generation”, where young learners are deeply immersed in a digitally rich environment, leading to the development of new learning styles and strategies. A study on effective human-computer interaction in digital academic supportive devices found that electronic academic devices in the learning environment provided a more attractive, realistic, and engaging teaching and learning experience [131]. Similarly, Kusumastuti et al. [132] revealed that incorporating digital devices in the classroom was associated with enhanced student motivation, improved application of course-based understanding, and overall academic achievement.

However, the impact of digital devices on academic performance is complex. A critical review of the literature on computer-based technology and student engagement highlights that the digital revolution has profoundly affected daily living, with mobile devices seamlessly integrated into common tasks [133]. This integration has presented unique opportunities and challenges for various industries, including education. As educational institutions strive to adapt to these technological advancements, it is crucial to examine how digital devices can be purposefully implemented to maximize student engagement and academic performance [134].

The integration of digital devices in the classroom has been shown to enhance the learning environment for all students. The use of electronic academic devices has been demonstrated to improve motivation, the ability to apply course-based understanding, and overall academic achievement [131]. Additionally, Bower et al. [135] established that categorizing academic digital devices into those that support the learning process and those that support the teaching process provides a framework for understanding the diverse applications of technology in education.

While the benefits of digital devices in academic settings are evident, it is essential to consider the potential drawbacks and implications. The ubiquity of technology has raised concerns about student engagement, as the seamless integration of devices into daily tasks may lead to distractions and reduced focus [133]. Thus, the impact of digital devices on academic performance is not without complexities. The effective use of human-computer interaction in digital academic supportive devices is crucial, as it can determine the level of engagement and learning outcomes [136]. Consequently, educators and institutions must navigate this delicate balance, leveraging the advantages of digital technology while mitigating the risks to ensure optimal learning outcomes.

In conclusion, the impact of digital devices on academic performance is a multifaceted and complex issue. Research highlights the potential of digital technologies to enhance learning, motivation, and higher-order thinking skills while also underscoring the need for careful consideration of the challenges and potential pitfalls [137]. As the digital landscape continues to evolve, educational stakeholders need to balance embracing technological advancements and maintaining a conducive learning environment that supports students’ holistic development.

### 2.10. Digital Devices and Psychological Development

The prevalence of digital device usage during early childhood is increasing. Consequently, there has been a growing interest in the impact of such usage on psychological development during this crucial period [138]. While this area of study is still developing, there are differing opinions within the scientific community. On one hand, some authors have praised the use of these devices as a means of learning, communication, and the development of essential skills, such as play, in children. On the other hand, some studies have presented evidence of the negative impact of these devices, linking their use to a higher prevalence of depressive symptoms, poorer sleep quality, a tendency towards hyperactivity, and problems with behavior and social interaction [139].

Recent studies suggested that during the first three years of life, when there is a high degree of brain plasticity, at least one third of children have access to a digital device such as a mobile phone or tablet [140]. Neuroplasticity enables children to learn and adapt to their environment during this period of their psychological development. Stimuli are essential for the production of synapses and the generation of functional structures in the brain. This constitutes the physiological basis for the psychological formations that shape the conditions for learning [141].

During childhood, a child’s brain is highly susceptible to the stimuli it receives from its immediate environment. Recent studies have shown that excessive use of mobile devices can lead to changes in the cerebral cortex, which can affect executive functions such as planning, working memory, and cognitive flexibility [140]. Therefore, it is essential to establish a maximum time limit for the use of mobile devices in children.

Additionally, the use of neuroimaging techniques to study the brains of children under the age of 12 has provided insight into the effects of digital activity on brain plasticity during critical periods of psychological development [142]. Alterations were identified in brain lobes such as the occipital lobe, which is involved in visual interpretation; the temporal lobe, which is involved in hearing, memory, and language processes; and the parietal lobe, which is involved in temperature regulation, pain, and touch processing. These are among the most important brain regions [143].

These studies suggested that the use of digital devices has a significant impact on the brain structure and functioning of children. However, the impact can be either positive or negative. In cases where the impact is negative, the use of digital devices is associated with poorer functioning of executive functions such as attention, inhibitory control, cognitive processes, and lower functional connectivity in brain areas associated with cognitive control and language [144]. Studies have shown that the use of digital devices, such as video games and the internet, can have a negative impact on brain volume and intelligence scores [145]. It is important to note that these findings are objective and not subjective evaluations.

Then again, a meta-analysis conducted by Bustamante et al. (2023) on the impact of screen time during early childhood and its correlation with executive functions found no significant effect on brain functioning. Notably, the studies analyzed may have yielded null results due to limitations in sample size or the focus of the study, which did not address the impact on brain development at the structural or functional level [146].

However, it has been reported by other authors that digital devices can have a beneficial effect and that video games can aid in skill development. For example, Mondéjar et al. (2016) conducted a study in which they found that the frontal lobe is significantly activated during the gaming phase. Using EEG/ERP, they observed that theta waves increased during the game, which improved learning and planning strategies. Regarding memory, the researchers observed an increase in beta waves when participants needed to recall information to advance in the game [147].

This line reports that previous studies have found that watching cartoons can enhance the activation of specific brain areas, including the bilateral medial temporal cortex, the superior parietal lobe, and the prefrontal cortex. Similarly, virtual reality has been linked to improvements in the activation of certain brain areas in the occipital and parietal lobes. These findings suggest that digital technologies can have a functional and structural impact on the brain’s lobes [112,148].

It is evident that there is still much to be studied regarding the effects of early digital device use on children’s psychological and cognitive development. Therefore, it is necessary to address this issue more comprehensively and systematically to gain a better understanding of its real impact.

### 2.11. Digital Devices and Unadaptative Behaviors

The scientific and educational community is studying disruptive behaviors associated with the use of digital devices, given their increasing availability from an early age [7]. Children acquire skills in accessing tablets, mobiles, computers, and other digital devices from the first months of life, making them vulnerable to certain content that they are exposed to on a recurring basis, often without adequate supervision by responsible adults.

The use of these devices as pedagogical tools can enhance learning skills and strategies. However, excessive use of these devices in the family context may lead to maladaptive behaviors that can negatively impact the maturational and social development of children and young people in today’s society [149].

**Figure 3:**
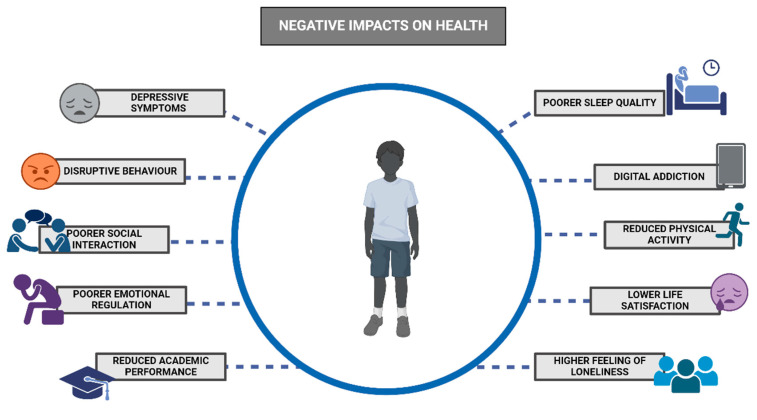
Potential Negative Impacts of Excessive Device Use on Children.

Defiant behavior is one of the most prevalent behaviors associated with excessive and uncontrolled use of digital devices. Such a negative behavior is determined by poor emotional regulation, which is learned and may be reinforced or punished through the use of digital devices in the family context, among other factors (Figure 3) [150].

Recent studies have shown that the use of digital devices, particularly mobiles and tablets, can cause significant behavioral changes. These changes not only affect the learning process and cognitive development but also lead to social isolation. Consequently, it reduces children’s and young people’s interest in participating in collective activities or practicing physical activities, which could have a negative impact on their relationships with peers [127].

Furthermore, it is noteworthy to mention the significant rise in the availability of these resources since the outbreak of the pandemic in 2020. Access to these resources increased due to the necessity of keeping schoolchildren connected. However, adequate strategies for proper monitoring and control of their use were not determined due to the immediate need to continue with academic and social life [151].

Recent studies have shown that the type of content children access on mobile devices has a significant impact on their behavior [152]. Specifically, it has been observed that children under the age of 6 exhibit more aggressive behavior when exposed to certain types of content, which can negatively affect their play behavior and other essential activities in the learning process [153].

Studies have also found negative results associated with the use of digital technologies from a very early age, including dependency tendencies that lead to persistent and obsessive use of mobile devices (Figure 3). It could result in symptoms such as anxiety, depression, and feelings of anguish, tension, and anger. It is important to note that these studies have shown a correlation, but not necessarily causation. This context enables disruptive behaviors that negatively impact the development of children and adolescents in family, educational, and social settings. These behaviors should be clearly marked as subjective evaluations if included [59].

During adolescence, negative, defiant, and hostile behaviors become increasingly prevalent. These behaviors can create a perception of an unsafe environment and lead to physical aggression, bullying, and a significant reduction in academic performance [115]. A study conducted by Kliesener et al. (2022) in Germany found that the use of smartphones among children and adolescents aged 12–18 years was linked to behavioral problems, impaired peer group interaction, and poor academic performance [154]. Studies conducted in India, the Czech Republic, Iran, and Spain have concluded that dependence on digital devices among children and adolescents is associated with behavioral problems, including irritability, anger, increased impulsivity, and disruptive behavior in academic and social contexts [155]. Furthermore, a direct correlation has been established between the excessive use of these devices and lower levels of life satisfaction, social support, and increased feelings of loneliness, stress, and depression (Figure 3) [156].

The use of digital devices in today’s society is a challenge for educators, families, and the scientific community. The data collected on their benefits and harms raises doubts among all actors involved in the education of children and young people worldwide. Therefore, it is crucial to establish policies that regulate the use of these resources in educational institutions. This will ensure that valuable resources are not wasted while also allowing for greater control over their content and usage time.

## 3. Practical Applications

Based on the provided data, here are practical applications for parents and schools to consider when integrating digital devices into children’s learning and development:

− Balanced Usage: Encourage a balanced approach to digital device usage, ensuring it does not replace physical activity, face-to-face interactions, and sufficient sleep.
− Content Selection: Curate educational content that aligns with your child’s age, interests, and learning objectives. Opt for apps and games that promote problem-solving, creativity, and memory skills.
− Parental Involvement: Actively participate in your child’s digital activities. Collaborative play and shared experiences with digital devices can enhance imaginative flexibility and social cognition.
− Monitoring and Guidance: Regularly monitor the type and amount of digital content consumed by your child. Guide them in using digital tools for learning rather than endless entertainment.
− Offline Enrichment: Complement digital learning with offline activities that reinforce digital literacy skills, such as reading, writing, and engaging in creative projects.
− Digital Hygiene: Teach children about digital hygiene, including the importance of taking breaks from screens and practicing good posture when using digital devices.
− Adaptive Learning: Implement adaptive learning technologies that tailor educational content to individual students’ needs, fostering a personalized learning experience.
− Integration of Digital Tools: Incorporate digital devices and educational software into the curriculum to enhance engagement and support diverse learning styles.
− Professional Development: Provide training for teachers on how to effectively use digital devices in the classroom, focusing on best practices and the latest educational technologies.
− Digital Citizenship: Educate students on digital citizenship, including online safety, responsible use of technology, and ethical behavior in the digital world.
− Blended Learning: Combine traditional teaching methods with digital resources to create blended learning environments that cater to different student needs and preferences.
− Assessment and Feedback: Utilize digital tools for formative assessments that provide immediate feedback, helping students track their progress and improve their learning outcomes.

Both parents and schools should aim to create a supportive environment that leverages the benefits of digital devices while being mindful of their potential drawbacks, ultimately ensuring that children’s cognitive, social, emotional, and psychological development is well-rounded and healthy.

## 4. Conclusions

Our narrative review has meticulously examined the multifaceted relationship between digital device usage and cognitive development in children, offering a comprehensive synthesis of the current knowledge in this domain. By conducting a rigorous literature search across various academic databases, the review has provided a robust analysis of how digital technologies can both enhance and impede cognitive abilities. It integrates psychological and neurophysiological perspectives to elucidate the mechanisms by which digital devices influence brain development, cognitive functions, and behavioral outcomes. Additionally, it underscores the complexity of the relationship, where factors such as age, gender, and socioeconomic status can significantly modulate the impact of digital device usage on cognitive development.

It has been highlighted that while digital devices can enhance executive functions such as cognitive control and decision-making, the effectiveness of such interventions is contingent upon the careful consideration of these modulating factors. Additionally, the integration of digital technology in educational settings has been shown to improve student engagement, motivation, and academic achievement. However, the ubiquity of digital devices also presents challenges, such as potential distractions and reduced focus, which can negatively impact academic performance. The negative consequences of excessive or inappropriate digital device usage include reduced attention spans and sleep disruptions. Also, the influence of digital devices on social cognition and social interaction is complex, with the potential for both positive outcomes, such as improved collaboration and empathy, and negative ones, such as social isolation and disruptive behaviors.

Furthermore, the review discusses the opportunities and challenges of digital device usage in fostering creativity and problem-solving skills. It suggests that while digital media can offer innovative learning experiences, excessive consumption can lead to adverse effects on physical health, cognitive abilities, and language development. It also emphasizes the importance of parental involvement and the quality of parent-child interactions in mediating the impact of digital devices on children’s cognitive and psychological development.

In conclusion, while digital devices offer numerous opportunities for cognitive enhancement and educational engagement, their use must be carefully managed to prevent adverse effects on children’s cognitive, psychological, and social development. Further research is needed to fully understand the long-term impacts of digital device usage and to inform the development of guidelines and policies that support the healthy integration of technology in children’s lives.

## Data Availability

Data are contained within the article.

## References

[1] Subrahmanyam K., Šmahel D. (2011). Connecting Online Behavior to Adolescent Development: A Theoretical Framework. Digital Youth: The Role of Media in Development.

[2] Valkenburg P.M., Piotrowski J.T. (2017). Plugged in: How Media Attract and Affect Youth.

[3] Lissak G. (2018). Adverse Physiological and Psychological Effects of Screen Time on Children and Adolescents: Literature Review and Case Study. Environ. Res..

[4] Volkow N.D., Wang G.-J., Fowler J.S., Tomasi D., Telang F. (2011). Addiction: Beyond Dopamine Reward Circuitry. Proc. Natl. Acad. Sci. USA.

[5] Green C.S., Bavelier D. (2012). Learning, Attentional Control, and Action Video Games. Curr. Biol. CB.

[6] Tap, Click, Read: Growing Readers in a World of Screens|Wiley. https://www.wiley.com/en-us/Tap%2C+Click%2C+Read%3A+Growing+Readers+in+a+World+of+Screens-p-9781119091899.

[7] Hill D., Ameenuddin N., Reid Chassiakos Y.L., Cross C., Hutchinson J., Levine A., Boyd R., Mendelson R., Moreno M., COUNCIL ON COMMUNICATIONS AND MEDIA (2016). Media and Young Minds. Pediatrics.

[8] Loh K.K., Kanai R. (2016). How Has the Internet Reshaped Human Cognition?. Neurosci. Rev. J. Bringing Neurobiol. Neurol. Psychiatry.

[9] Dux P.E., Tombu M.N., Harrison S., Rogers B.P., Tong F., Marois R. (2009). Training Improves Multitasking Performance by Increasing the Speed of Information Processing in Human Prefrontal Cortex. Neuron.

[10] Cognitive Load During Problem Solving: Effects on Learning—Sweller—1988—Cognitive Science—Wiley Online Library. https://onlinelibrary.wiley.com/doi/abs/10.1207/s15516709cog1202_4.

[11] Miyake A., Friedman N.P., Emerson M.J., Witzki A.H., Howerter A., Wager T.D. (2000). The Unity and Diversity of Executive Functions and Their Contributions to Complex “Frontal Lobe” Tasks: A Latent Variable Analysis. Cognit. Psychol..

[12] Kostyrka-Allchorne K., Cooper N.R., Simpson A. (2017). The Relationship between Television Exposure and Children’s Cognition and Behaviour: A Systematic Review. Dev. Rev..

[13] Ophir E., Nass C., Wagner A.D. (2009). Cognitive Control in Media Multitaskers. Proc. Natl. Acad. Sci. USA.

[14] Greenfield P.M. (2009). Technology and Informal Education: What Is Taught, What Is Learned. Science.

[15] Koepp M.J., Gunn R.N., Lawrence A.D., Cunningham V.J., Dagher A., Jones T., Brooks D.J., Bench C.J., Grasby P.M. (1998). Evidence for Striatal Dopamine Release during a Video Game. Nature.

[16] Spence A., Beasley K., Gravenkemper H., Hoefler A., Ngo A., Ortiz D., Campisi J. (2020). Social Media Use While Listening to New Material Negatively Affects Short-Term Memory in College Students. Physiol. Behav..

[17] Piaget J. (1952). The Origins of Intelligence in Children.

[18] Estévez B., Gabriela M. (2022). Teoría Psicogenética de Jean Piaget: Aportes Para Comprender al Niño de Hoy Que Será El Adulto Del Mañana.

[19] Madigan S., Browne D., Racine N., Mori C., Tough S. (2019). Association Between Screen Time and Children’s Performance on a Developmental Screening Test. JAMA Pediatr..

[20] Huber B., Yeates M., Meyer D., Fleckhammer L., Kaufman J. (2018). The Effects of Screen Media Content on Young Children’s Executive Functioning. J. Exp. Child Psychol..

[21] Mind in Society. https://www.hup.harvard.edu/books/9780674576292.

[22] Przybylski A.K., Weinstein N. (2017). A Large-Scale Test of the Goldilocks Hypothesis: Quantifying the Relations between Digital-Screen Use and the Mental Well-Being of Adolescents. Psychol. Sci..

[23] Twenge J.M., Campbell W.K. (2018). Associations between Screen Time and Lower Psychological Well-Being among Children and Adolescents: Evidence from a Population-Based Study. Prev. Med. Rep..

[24] Spence I., Feng J. (2010). Video Games and Spatial Cognition. https://journals.sagepub.com/doi/10.1037/a0019491.

[25] Uttal D.H., Meadow N.G., Tipton E., Hand L.L., Alden A.R., Warren C., Newcombe N.S. (2013). The Malleability of Spatial Skills: A Meta-Analysis of Training Studies. Psychol. Bull..

[26] Cheng K.-H., Tsai C.-C. (2013). Affordances of Augmented Reality in Science Learning: Suggestions for Future Research. J. Sci. Educ. Technol..

[27] Clements D.H., Sarama J. (2011). Early Childhood Mathematics Intervention. Science.

[28] Hutton J.S., Horowitz-Kraus T., Mendelsohn A.L., DeWitt T., Holland S.K. (2015). C-MIND Authorship Consortium Home Reading Environment and Brain Activation in Preschool Children Listening to Stories. Pediatrics.

[29] Franceschini S., Gori S., Ruffino M., Viola S., Molteni M., Facoetti A. (2013). Action Video Games Make Dyslexic Children Read Better. Curr. Biol. CB.

[30] Beaumont R., Sofronoff K. (2008). A Multi-Component Social Skills Intervention for Children with Asperger Syndrome: The Junior Detective Training Program. J. Child Psychol. Psychiatry.

[31] Neumann M.M., Neumann D.L. (2017). The Use of Touch-Screen Tablets at Home and Pre-School to Foster Emergent Literacy. https://journals.sagepub.com/doi/10.1177/1468798415619773.

[32] Personalized Adaptive Learning: An Emerging Pedagogical Approach Enabled by a Smart Learning Environment|Smart Learning Environments. https://link.springer.com/article/10.1186/s40561-019-0089-y.

[33] Weiss P.L., Rand D., Katz N., Kizony R. (2004). Video Capture Virtual Reality as a Flexible and Effective Rehabilitation Tool. J. Neuroeng. Rehabil..

[34] Reich S.M., Yau J.C., Warschauer M. (2016). Tablet-Based eBooks for Young Children: What Does the Research Say?. J. Dev. Behav. Pediatr. JDBP.

[35] Digital Youth: The Role of Media in Development|SpringerLink. https://link.springer.com/book/10.1007/978-1-4419-6278-2.

[36] Granic I., Lobel A., Engels R.C.M.E. (2014). The Benefits of Playing Video Games. Am. Psychol..

[37] Jordan T. (2009). The Ecology of Games: Connecting Youth, Games and Learning. Inf. Commun. Soc..

[38] Khatib F., Cooper S., Tyka M.D., Xu K., Makedon I., Popović Z., Baker D. (2011). Algorithm Discovery by Protein Folding Game Players. PNAS Proc. Natl. Acad. Sci. USA.

[39] Dye M.W.G., Green C.S., Bavelier D. (2009). Increasing Speed of Processing With Action Video Games. Curr. Dir. Psychol. Sci..

[40] Bavelier D., Green C.S., Pouget A., Schrater P. (2012). Brain Plasticity through the Life Span: Learning to Learn and Action Video Games. Annu. Rev. Neurosci..

[41] Kollins S.H., DeLoss D.J., Cañadas E., Lutz J., Findling R.L., Keefe R.S.E., Epstein J.N., Cutler A.J., Faraone S.V. (2020). A Novel Digital Intervention for Actively Reducing Severity of Paediatric ADHD (STARS-ADHD): A Randomised Controlled Trial. Lancet Digit. Health.

[42] Alloway T.P., Alloway R.G. (2010). Investigating the Predictive Roles of Working Memory and IQ in Academic Attainment. J. Exp. Child Psychol..

[43] Mayer E.A. (2011). Gut Feelings: The Emerging Biology of Gut-Brain Communication. Nat. Rev. Neurosci..

[44] Peppler K.A., Kafai Y.B. (2007). From SuperGoo to Scratch: Exploring Creative Digital Media Production in Informal Learning. Learn. Media Technol..

[45] Jackson L.A., Witt E.A., Games A.I., Fitzgerald H.E., von Eye A., Zhao Y. (2012). Information Technology Use and Creativity: Findings from the Children and Technology Project. Comput. Hum. Behav..

[46] Children and Adolescents and Digital Media|Pediatrics|American Academy of Pediatrics. https://publications.aap.org/pediatrics/article/138/5/e20162593/60349/Children-and-Adolescents-and-Digital-Media.

[47] Chun M.M., Golomb J.D., Turk-Browne N.B. (2011). A Taxonomy of External and Internal Attention. Annu. Rev. Psychol..

[48] Oberauer K. (2019). Working Memory and Attention—A Conceptual Analysis and Review. J. Cogn..

[49] Carrera B., Mazzarella C. (2001). Vygotsky: Enfoque Sociocultural. Educere.

[50] Green C.S., Bavelier D. (2003). Action Video Game Modifies Visual Selective Attention. Nature.

[51] Rosen L.D. (2010). Rewired: Understanding the iGeneration and the Way They Learn. Educ. Dig. Essent. Read. Condens. Quick Rev..

[52] Pham H.T., Chuang H.-L., Kuo C.-P., Yeh T.-P., Liao W.-C. (2021). Electronic Device Use before Bedtime and Sleep Quality among University Students. Healthcare.

[53] Muennig P., Schweinhart L., Montie J., Neidell M. (2009). Effects of a Prekindergarten Educational Intervention on Adult Health: 37-Year Follow-Up Results of a Randomized Controlled Trial. Am. J. Public Health.

[54] Hale L., Guan S. (2015). Screen Time and Sleep among School-Aged Children and Adolescents: A Systematic Literature Review. Sleep Med. Rev..

[55] Twenge J.M., Campbell W.K. (2019). Media Use Is Linked to Lower Psychological Well-Being: Evidence from Three Datasets. Psychiatr. Q..

[56] Radesky J.S., Christakis D.A. (2016). Increased Screen Time: Implications for Early Childhood Development and Behavior. Pediatr. Clin. N. Am..

[57] LeBourgeois M.K., Hale L., Chang A.-M., Akacem L.D., Montgomery-Downs H.E., Buxton O.M. (2017). Digital Media and Sleep in Childhood and Adolescence. Pediatrics.

[58] Carter B., Rees P., Hale L., Bhattacharjee D., Paradkar M.S. (2016). Association Between Portable Screen-Based Media Device Access or Use and Sleep Outcomes: A Systematic Review and Meta-Analysis. JAMA Pediatr..

[59] Anderson D.R., Subrahmanyam K. (2017). Cognitive Impacts of Digital Media Workgroup Digital Screen Media and Cognitive Development. Pediatrics.

[60] Straker L., Pollock C., Maslen B. (2009). Principles for the Wise Use of Computers by Children. Ergonomics.

[61] Lauricella A.R., Wartella E.A., Rideout V.J. (2015). Young Children’s Screen Time: The Complex Role of Parent and Child Factors. J. Appl. Dev. Psychol..

[62] Joan Ganz Cooney Center—Always Connected: The New Digital Media Habits of Young Children. https://joanganzcooneycenter.org/publication/always-connected-the-new-digital-media-habits-of-young-children/.

[63] Veraksa N., Veraksa A., Gavrilova M., Bukhalenkova D., Oshchepkova E., Chursina A. (2021). Short- and Long-Term Effects of Passive and Active Screen Time on Young Children’s Phonological Memory. Front. Educ..

[64] Papadakis S., Zaranis N., Kalogiannakis M. (2019). Parental Involvement and Attitudes towards Young Greek Children’s Mobile Usage. Int. J. Child-Comput. Interact..

[65] Morehead K., Dunlosky J., Rawson K.A. (2019). How Much Mightier Is the Pen than the Keyboard for Note-Taking? A Replication and Extension of Mueller and Oppenheimer (2014). Educ. Psychol. Rev..

[66] Mayer R.E. Multimedia Learning. https://www.cambridge.org/highereducation/books/multimedia-learning/FB7E79A165D24D47CEACEB4D2C426ECD.

[67] Uncapher M.R., Wagner A.D. (2018). Minds and Brains of Media Multitaskers: Current Findings and Future Directions. Proc. Natl. Acad. Sci. USA.

[68] Ward A.F., Duke K., Gneezy A.Y., Bos M.W. (2017). Brain Drain: The Mere Presence of One’s Own Smartphone Reduces Available Cognitive Capacity. J. Assoc. Consum. Res..

[69] Carr N. (2010). The Shallows: What The Internet Is Doing to Our Brains.

[70] Sparrow B., Liu J., Wegner D.M. (2011). Google Effects on Memory: Cognitive Consequences of Having Information at Our Fingertips. Science.

[71] Diamond A. (2013). Executive Functions. Annu. Rev. Psychol..

[72] Fernández M.M.O., Ferreiro A.A. (2023). Las tecnologías digitales en el entrenamiento de las funciones ejecutivas: Una revisión sistemática de literatura. RiiTE Rev. Interuniv. Investig. Tecnol. Educ..

[73] Mendoza L.R.M., Martínez M.E.M. (2020). TIC y neuroeducación como recurso de innovación en el proceso de enseñanza y aprendizaje. ReHuSo Rev. Cienc. Humanísticas Soc..

[74] Pérez Fernández C., Cánovas R., Moreno-Montoya M., Sánchez F., Flores Cubos P. (2017). Go/NoGo training improves executive functions in an 8-year-old child born preterm. Rev. Psicol. Clínica Con. Niños Adolesc..

[75] Ramos D.K., Garcia F.A. (2019). Digital Games and Improvement of the Inhibitory Control: A Study With Children in Specialized Educational Service. Rev. Bras. Educ. Espec..

[76] Najberg H., Wachtl L., Anziano M., Mouthon M., Spierer L. (2021). Aging Modulates Prefrontal Plasticity Induced by Executive Control Training. Cereb. Cortex.

[77] Russo-Johnson C., Troseth G., Duncan C., Mesghina A. (2017). All Tapped Out: Touchscreen Interactivity and Young Children’s Word Learning. Front. Psychol..

[78] Healey A., Mendelsohn A., Sells J.M., Donoghue E., Earls M., Hashikawa A., McFadden T., Peacock G., Scholer S., COUNCIL ON EARLY CHILDHOOD (2019). Selecting Appropriate Toys for Young Children in the Digital Era. Pediatrics.

[79] Thagard P., Stewart T.C. (2011). The AHA! Experience: Creativity through Emergent Binding in Neural Networks. Cogn. Sci..

[80] Gaspar D., Mabic M. (2015). Creativity in Higher Education. Univers. J. Educ. Res..

[81] Lin Y. (2014). A Third Space for Dialogues on Creative Pedagogy: Where Hybridity Becomes Possible. Think. Ski. Creat..

[82] Khalaf S., Kilani H., Razo M.B., Grigorenko E.L. (2022). Bored, Distracted, and Confused: Emotions That Promote Creativity and Learning in a 28-Month-Old Child Using an iPad. J. Intell..

[83] Felix E., Silva V., Caetano M., Ribeiro M.V.V., Fidalgo T.M., Rosa Neto F., Sanchez Z.M., Surkan P.J., Martins S.S., Caetano S.C. (2020). Excessive Screen Media Use in Preschoolers Is Associated with Poor Motor Skills. Cyberpsychol. Behav. Soc. Netw..

[84] Oswald T.K., Rumbold A.R., Kedzior S.G.E., Moore V.M. (2020). Psychological Impacts of “Screen Time” and “Green Time” for Children and Adolescents: A Systematic Scoping Review. PLoS ONE.

[85] Ishii K., Shibata A., Koohsari M.J., Oka K. (2022). The Relationships between Parents’ and Children’s Screen Times on Body Mass Index: A Cross-Sectional Path Analysis. BMC Public Health.

[86] Qu G., Hu W., Meng J., Wang X., Su W., Liu H., Ma S., Sun C., Huang C., Lowe S. (2023). Association between Screen Time and Developmental and Behavioral Problems among Children in the United States: Evidence from 2018 to 2020 NSCH. J. Psychiatr. Res..

[87] Bukhalenkova D., Almazova O. (2023). Active Screen Time and Imagination in 5–6-Years-Old Children. Front. Psychol..

[88] Putnam A.L. (2015). Mnemonics in Education: Current Research and Applications. Transl. Issues Psychol. Sci..

[89] Herman W.E. (1991). Creativity: A Missing Pedagogical Link for the Preparation of Teachers. Teach. Educ..

[90] Cioca L.-I., Nerișanu R.A. (2020). Enhancing Creativity: Using Visual Mnemonic Devices in the Teaching Process in Order to Develop Creativity in Students. Sustainability.

[91] Heard M.M. (2017). Examining the Use of Mnemonic Devices in Instructional Practices to Improve the Reading Skills of Third Grade Public School Students with Learning Disabilities. Ph.D. Dissertation.

[92] Pillai N.R. (2004). Using Mnemonics to Improve Vocabulary, Boost Memory and Enhance Creativity in the ESL Classroom. Engl. Teach..

[93] Liao Y.-H., Kung W.-C., Chen H.-C. (2019). Testing the Effectiveness of Creative Map Mnemonic Strategies in a Geography Class. Instr. Sci..

[94] Latiff N., Najmi M.H.S.M., Rouyan N. (2016). A Case Study on the Effects of Mnemonics on English Vocabulary. Int. J. Appl. Linguist. Engl. Lit..

[95] Hill A.C. (2022). The Effectiveness of Mnemonic Devices for ESL Vocabulary Retention. Engl. Lang. Teach..

[96] Knote R., Janson A., Söllner M., Leimeister J.M. (2020). Value Co-Creation in Smart Services: A Functional Affordances Perspective on Smart Personal Assistants. J. Assoc. Inf. Syst..

[97] Winkler R., Söllner M., Leimeister J.M. (2021). Enhancing Problem-Solving Skills with Smart Personal Assistant Technology. Comput. Educ..

[98] Winkler R., Neuweiler M.L., Bittner E.A., Söllner M. Hey Alexa, Please Help Us Solve This Problem! How Interactions with Smart Personal Assistants Improve Group Performance. Proceedings of the 40th International Conference on Information Systems, ICIS 2019.

[99] Winkler R., Söllner M. Towards Empowering Educators to Create Their Own Smart Personal Assistants. Proceedings of the 53rd Annual Hawaii International Conference on System Sciences.

[100] Shaikh S.J., Cruz I.F. (2023). AI in Human Teams: Effects on Technology Use, Members’ Interactions, and Creative Performance under Time Scarcity. AI Soc..

[101] Reddy A. (2022). Artificial Everyday Creativity: Creative Leaps with AI through Critical Making. Digit. Creat..

[102] Bown O. (2021). Beyond the Creative Species: Making Machines That Make Art and Music.

[103] Vinchon F., Lubart T., Bartolotta S., Gironnay V., Botella M., Bourgeois-Bougrine S., Burkhardt J.-M., Bonnardel N., Corazza G.E., Glăveanu V. (2023). Artificial Intelligence & Creativity: A Manifesto for Collaboration. J. Creat. Behav..

[104] Ameen N., Sharma G.D., Tarba S., Rao A., Chopra R. (2022). Toward Advancing Theory on Creativity in Marketing and Artificial Intelligence. Psychol. Mark..

[105] Marrone R., Taddeo V., Hill G. (2022). Creativity and Artificial Intelligence—A Student Perspective. J. Intell..

[106] Creely E., Henriksen D., Henderson M. (2023). Artificial Intelligence, Creativity, and Education: Critical Questions for Researchers and Educators.

[107] Pérez A.G., Díaz M.J.S. (2021). Aspectos pedagógicos, tecnológicos y de interacción social del aprendizaje móvil: Revisión Sistemática de Literatura. Educ. Siglo XXI.

[108] Beaudoin C., Beauchamp M.H. (2020). Social Cognition. Handb. Clin. Neurol..

[109] Lin L.-Y., Cherng R.-J., Chen Y.-J., Chen Y.-J., Yang H.-M. (2015). Effects of Television Exposure on Developmental Skills among Young Children. Infant Behav. Dev..

[110] Tomopoulos S., Dreyer B.P., Berkule S., Fierman A.H., Brockmeyer C., Mendelsohn A.L. (2010). Infant Media Exposure and Toddler Development. Arch. Pediatr. Adolesc. Med..

[111] Christakis D.A., Garrison M.M., Herrenkohl T., Haggerty K., Rivara F.P., Zhou C., Liekweg K. (2013). Modifying Media Content for Preschool Children: A Randomized Controlled Trial. Pediatrics.

[112] Hutton J.S., Dudley J., Horowitz-Kraus T., DeWitt T., Holland S.K. (2020). Associations Between Screen-Based Media Use and Brain White Matter Integrity in Preschool-Aged Children. JAMA Pediatr..

[113] Ortega Mohedano F., Pinto Hernández F. (2021). Predicción del bienestar sobre el uso de pantallas inteligentes de los niños. Comun. Rev. Científica Comun. Educ..

[114] Cadena K.A.Á., Alvarado K.G.M., Asacata D.E.P., Chong M.K.N.K. (2020). Tiempo en pantalla (televisión, computadora, celular, tabletas) en las relaciones interpersonales entre niños de 8 a 12 años. Horiz. Rev. Investig. En Cienc. Educ..

[115] Guzmán-Brand V., Gelvez-García L. (2022). Phubbing en los adolescentes un comportamiento que afecta la interacción social. Una revisión sistemática. Rev. Estud. Psicológicos.

[116] Gómez R.S., Lanna L.C., Oro M.G.I. (2013). Análisis del entorno colaborativo creado para una experiencia de mobile learning. Educ. Knowl. Soc. EKS.

[117] Brown D., Lamb M. (2022). Digital Temperance: Adapting an Ancient Virtue for a Technological Age. Ethics Inf. Technol..

[118] Bhat R. (2023). The Impact of Technology Integration on Student Learning Outcomes: A Comparative Study. Int. J. Soc. Sci. Educ. Econ. Agric. Res. Technol. IJSET.

[119] Hiustra M.L., Angelica A.V., Pangaribuan C.H. (2023). Social Media’s Negative Impact on Mental Health Subjugated by the Advantage on Young Adults. Bus. Econ. Commun. Soc. Sci. J. BECOSS.

[120] Khalaf A.M., Alubied A.A., Khalaf A.M., Rifaey A.A. (2023). The Impact of Social Media on the Mental Health of Adolescents and Young Adults: A Systematic Review. Cureus.

[121] da Silva M.P.F.N., Cardoso G.M. (2023). da S.; Priolo Filho, S.R.; Weber, S.A.T.; Corrêa, C. de C. Technologies and Mental Health in University Students: An Unhealthy Combination. Int. Arch. Otorhinolaryngol..

[122] Gupta C., Jogdand D.S., Kumar M. (2022). Reviewing the Impact of Social Media on the Mental Health of Adolescents and Young Adults. Cureus.

[123] Cain J. (2018). It’s Time to Confront Student Mental Health Issues Associated with Smartphones and Social Media. Am. J. Pharm. Educ..

[124] Michael S.L., Jones S.E., Merlo C.L., Sliwa S.A., Lee S.M., Cornett K., Brener N.D., Chen T.J., Ashley C.L., Park S. (2023). Dietary and Physical Activity Behaviors in 2021 and Changes from 2019 to 2021 Among High School Students—Youth Risk Behavior Survey, United States, 2021. MMWR Suppl..

[125] Orzech K.M., Grandner M.A., Roane B.M., Carskadon M.A. (2016). Digital Media Use in the 2 h before Bedtime Is Associated with Sleep Variables in University Students. Comput. Hum. Behav..

[126] Sutikno H., Basit A. (2023). The Impact of Social Media Use on Social Interaction of Students of The Faculty of Medicine, Jenderal Soedirman University. Int. J. Soc. Sci. Educ. Commun. Econ. SINOMICS J..

[127] Nakshine V.S., Thute P., Khatib M.N., Sarkar B. (2022). Increased Screen Time as a Cause of Declining Physical, Psychological Health, and Sleep Patterns: A Literary Review. Cureus.

[128] Mireku M.O., Barker M.M., Mutz J., Shen C., Dumontheil I., Thomas M.S.C., Röösli M., Elliott P., Toledano M.B. (2019). Processed Data on the Night-Time Use of Screen-Based Media Devices and Adolescents’ Sleep Quality and Health-Related Quality of Life. Data Brief.

[129] Kim H.J., Yi P., Hong J.I. (2020). Students’ Academic Use of Mobile Technology and Higher-Order Thinking Skills: The Role of Active Engagement. Educ. Sci..

[130] Heflin H., Shewmaker J., Nguyen J. (2017). Impact of Mobile Technology on Student Attitudes, Engagement, and Learning. Comput. Educ..

[131] Thuseethan S., Kuhanesan S. (2014). Effective Use of Human Computer Interaction in Digital Academic Supportive Devices. Int. J. Sci. Res. IJSR.

[132] Kusumastuti D.L., Tjhin V., Soraya K. The Role of Mobile Devices to Improve Student Learning Motivation on Distance Learning. Proceedings of the 2017 International Conference on Information Technology.

[133] Schindler L.A., Burkholder G.J., Morad O.A., Marsh C. (2017). Computer-Based Technology and Student Engagement: A Critical Review of the Literature. Int. J. Educ. Technol. High. Educ..

[134] Garlinska M., Osial M., Proniewska K., Pregowska A. (2023). The Influence of Emerging Technologies on Distance Education. Electronics.

[135] Bower M., Wood L., Lai J., Howe C., Lister R., Mason R., Highfield K., Veal J. (2017). Improving the Computational Thinking Pedagogical Capabilities of School Teachers. Aust. J. Teach. Educ..

[136] Rashid T., Asghar H.M. (2016). Technology Use, Self-Directed Learning, Student Engagement and Academic Performance: Examining the Interrelations. Comput. Hum. Behav..

[137] Murshidi G.A. (2017). Opportunities and Challenges of Mobile Learning That University Students Encounter in the UAE. Int. Res. High. Educ..

[138] Cao S., Li H. (2023). A Scoping Review of Digital Well-Being in Early Childhood: Definitions, Measurements, Contributors, and Interventions. Int. J. Environ. Res. Public. Health.

[139] Chaarani B., Ortigara J., Yuan D., Loso H., Potter A., Garavan H.P. (2022). Association of Video Gaming With Cognitive Performance Among Children. JAMA Netw. Open.

[140] Helsper E.J., Eynon R. (2010). Digital Natives: Where Is the Evidence?. Br. Educ. Res. J..

[141] Braune-Krickau K., Schneebeli L., Pehlke-Milde J., Gemperle M., Koch R., von Wyl A. (2021). Smartphones in the Nursery: Parental Smartphone Use and Parental Sensitivity and Responsiveness within Parent-Child Interaction in Early Childhood (0–5 Years): A Scoping Review. Infant Ment. Health J..

[142] Korte M. (2020). The Impact of the Digital Revolution  on Human Brain and Behavior: Where  Do We Stand?. Dialogues Clin. Neurosci..

[143] How Early Digital Experience Shapes Young Brains During 0–12 Years: A Scoping Review: Early Education and Development. https://www.tandfonline.com/doi/full/10.1080/10409289.2023.2278117.

[144] Law E.C., Han M.X., Lai Z., Lim S., Ong Z.Y., Ng V., Gabard-Durnam L.J., Wilkinson C.L., Levin A.R., Rifkin-Graboi A. (2023). Associations Between Infant Screen Use, Electroencephalography Markers, and Cognitive Outcomes. JAMA Pediatr..

[145] Matsuda G., Hiraki K. (2006). Sustained Decrease in Oxygenated Hemoglobin during Video Games in the Dorsal Prefrontal Cortex: A NIRS Study of Children. NeuroImage.

[146] Bustamante J.C., Fernández-Castilla B., Alcaraz-Iborra M. (2023). Relation between Executive Functions and Screen Time Exposure in under 6 Year-Olds: A Meta-Analysis. Comput. Hum. Behav..

[147] Mondéjar T., Hervás R., Johnson E., Gutierrez C., Latorre J.M. (2016). Correlation between Videogame Mechanics and Executive Functions through EEG Analysis. J. Biomed. Inform..

[148] Hutton J.S., Dudley J., Horowitz-Kraus T., DeWitt T., Holland S.K. (2020). Differences in Functional Brain Network Connectivity during Stories Presented in Audio, Illustrated, and Animated Format in Preschool-Age Children. Brain Imaging Behav..

[149] Nathanson A.I., Fries P.T. (2014). Television Exposure, Sleep Time, and Neuropsychological Function Among Preschoolers. Media Psychol..

[150] Arabiat D., Al Jabery M., Robinson S., Whitehead L., Mörelius E. (2023). Interactive Technology Use and Child Development: A Systematic Review. Child Care Health Dev..

[151] Mesce M., Ragona A., Cimino S., Cerniglia L. (2022). The Impact of Media on Children during the COVID-19 Pandemic: A Narrative Review. Heliyon.

[152] Pea R., Nass C., Meheula L., Rance M., Kumar A., Bamford H., Nass M., Simha A., Stillerman B., Yang S. (2012). Media Use, Face-to-Face Communication, Media Multitasking, and Social Well-Being among 8- to 12-Year-Old Girls. Dev. Psychol..

[153] Yoon S., Kleinman M., Mertz J., Brannick M. (2019). Is Social Network Site Usage Related to Depression? A Meta-Analysis of Facebook-Depression Relations. J. Affect. Disord..

[154] Kliesener T., Meigen C., Kiess W., Poulain T. (2022). Associations between Problematic Smartphone Use and Behavioural Difficulties, Quality of Life, and School Performance among Children and Adolescents. BMC Psychiatry.

[155] Menéndez-García A., Jiménez-Arroyo A., Rodrigo-Yanguas M., Marin-Vila M., Sánchez-Sánchez F., Roman-Riechmann E., Blasco-Fontecilla H. (2020). Adicción a Internet, Videojuegos y Teléfonos Móviles En Niños y Adolescentes: Un Estudio de Casos y Controles. Adicciones.

[156] Nikolic A., Bukurov B., Kocic I., Vukovic M., Ladjevic N., Vrhovac M., Pavlović Z., Grujicic J., Kisic D., Sipetic S. (2023). Smartphone Addiction, Sleep Quality, Depression, Anxiety, and Stress among Medical Students. Front. Public Health.

